# From Molecular Mechanisms to Clinical Strategies: A Comprehensive Overview of Metabolic Dysfunction-Associated Steatotic Liver Disease (MASLD)

**DOI:** 10.3390/ijms27177825

**Published:** 2026-09-01

**Authors:** Damian Świerczek, Maja Dreger, Jakub Jatkowski, Jakub Kancerek, Bogna Drozdzowska, Romuald Wojnicz

**Affiliations:** 1 Department of Histology and Cell Pathology, Faculty of Medical Sciences in Zabrze, Medical University of Silesia in Katowice, 40-055 Katowice, Poland; s88516@365.sum.edu.pl (D.Ś.); s92054@365.sum.edu.pl (M.D.); s92037@365.sum.edu.pl (J.J.); s88540@365.sum.edu.pl (J.K.); 2 Department and Pathomorphology Division, Faculty of Medical Sciences in Zabrze, Medical University of Silesia in Katowice, 40-055 Katowice, Poland; bdrozdzowska@sum.edu.pl

**Keywords:** MASLD, chronic liver disease, multiple-hit hypothesis, clinical course, multiparametric imaging, treatment

## Abstract

Metabolic dysfunction-associated steatotic liver disease (MASLD), formerly known as non-alcoholic fatty liver disease (NAFLD), has become one of the most common chronic liver diseases worldwide, representing a major global health challenge closely linked to metabolic syndrome, obesity, and type 2 diabetes mellitus (TD2M). The condition is characterized by a multisystem nature driven by complex multifactorial mechanisms, including insulin resistance, lipotoxicity, mitochondrial dysfunction, genetic predispositions, and gut-liver axis alterations. Although liver biopsy remains the gold standard, non-invasive markers and advanced imaging methods are of key importance for early risk stratification. Furthermore, while liver-related complications such as fibrosis, cirrhosis, and hepatocellular carcinoma (HCC) are significant, cardiovascular disease remains the leading cause of mortality in patients with MASLD. Current management relies primarily on lifestyle modifications, targeted pharmacotherapy (such as pioglitazone, GLP-1 receptor agonists, SGLT-2 inhibitors), and novel experimental therapies. Consequently, MASLD requires a multidisciplinary approach emphasizing early diagnosis, risk stratification, and comprehensive treatment of both hepatic and extrahepatic manifestations.

## 1. Introduction

Metabolic dysfunction-associated steatotic liver disease (MASLD), formerly known as non-alcoholic fatty liver disease (NAFLD), is currently one of the most common chronic liver diseases worldwide [1,2]. The adoption of the overarching steatotic liver disease nomenclature and updated MASLD criteria was aimed at eliminating stigmatizing language and diagnoses of exclusion in favor of explicit cardiometabolic criteria [3]. It encompasses a broad spectrum of clinical manifestations, ranging from simple steatosis and metabolic dysfunction-associated steatohepatitis (MASH) to progression toward liver cirrhosis and hepatocellular carcinoma (HCC). Current data indicate a rapid rise in the global burden of MASLD. It is estimated that MASLD currently affects approximately 38% of the global adult population, representing a 50% increase compared to the period from 1990 to 2006. This trend is expected to persist, with global prevalence among adults projected to exceed 55% by 2040. Significant geographical variations exist, with the highest prevalence recorded in Latin America (44.4%) and the lowest in Western Europe (25.1%). Incidence rates are higher in men across all age groups, peaking at 80–84 years of age for both sexes before declining. Meanwhile, the absolute number of MASLD cases is highest among younger adults, peaking at ages 35–39 in men and 55–59 in women [1,2]. A significant clinical challenge is that the early stages of MASLD are usually asymptomatic, making early diagnosis difficult. Consequently, non-invasive diagnostic methods, such as liver enzyme measurements and fibrosis assessment scores, are widely used in clinical practice as screening, diagnostic, and prognostic tools [4,5]. Diagnostic evaluation is further supported by imaging techniques, which play a crucial role in assessing hepatic steatosis and the risk of disease progression. In particular, modern multiparametric imaging modalities have gained importance due to their higher sensitivity compared with conventional imaging methods. Despite substantial advances in non-invasive diagnostics, liver biopsy remains the gold standard for assessing disease severity; however, its use is limited by its invasive nature and the risk of complications. According to current guidelines, the diagnosis of MASLD is based on the presence of hepatic steatosis detected by imaging or histopathological examination after excluding other significant causes of steatosis. In clinical practice, alcohol consumption is also considered and should not exceed 20 g/day in women and 30 g/day in men. In addition, at least one criterion of metabolic dysfunction must be fulfilled to establish the diagnosis [6,7,8].

MASLD is strongly associated with metabolic disorders, particularly metabolic syndrome, which is diagnosed in approximately 43% of patients. The prevalence of MASLD among individuals who are overweight and obese is approximately 70% and 75%, respectively, while among patients with type 2 diabetes, it reaches 68.8%. Furthermore, insulin resistance occurs in approximately 80% of individuals with MASLD, whereas hypertension is present in 40–60% of patients. Although the risk of advanced fibrosis and progression to hepatocellular carcinoma remains relatively low, individuals with MASLD are more likely to die from cardiovascular causes than from liver-related complications [1,2,3]. Therefore, given the metabolic basis of the disease, the management of MASLD primarily focuses on lifestyle modification, including weight reduction, adherence to the Mediterranean diet, and increased physical activity. Pharmacological treatment is mainly aimed at improving metabolic parameters, including glucose and lipid metabolism, and its selection depends on the presence of obesity, type 2 diabetes mellitus, and cardiovascular risk factors. In advanced stages of the disease, management of cirrhosis-related complications may be required, and liver transplantation should be considered in selected cases [9,10]. Given the growing epidemiological significance and complex nature of MASLD, the aim of this review was to systematize the current knowledge regarding this disease entity, with particular emphasis on its etiopathogenesis, diagnostics, clinical course, histopathological features, and available as well as emerging therapeutic strategies, while also highlighting directions for future research.

## 2. Disease Progression and Clinical Outcomes

MASLD is often asymptomatic or presents with non-specific symptoms such as fatigue and a tendency for right upper quadrant discomfort. This leads to delayed diagnosis, which frequently occurs only during the evaluation of coexisting metabolic disorders [11]. Contrary to expectations, cardiovascular disease is the primary cause of death in patients with MASLD, highlighting the profound systemic impact of steatosis-driven endothelial dysfunction [12]. Epidemiological data indicate that against an all-cause mortality rate of 12.60 per 1000 person-years (PY), the pooled cardiac-specific mortality rate is 4.20 per 1000 PY. Extrahepatic malignancies represent the second most common cause of death (2.83 per 1000 PY), followed by liver-related complications (0.92 per 1000 PY), including decompensated cirrhosis and hepatocellular carcinoma [11,13,14]. Progression from isolated steatosis to MASH and fibrosis is highly variable. Accelerating factors include advanced age, visceral obesity, and type 2 diabetes mellitus (T2DM) [15]. Genetic variants, particularly *PNPLA3 I148M*, act as independent drivers [16], while hormonal status strongly modulates this risk, as premenopausal women show lower susceptibility than men, but the menopause-related decline in estrogen levels significantly accelerates disease progression [17,18].

**Figure 1:**
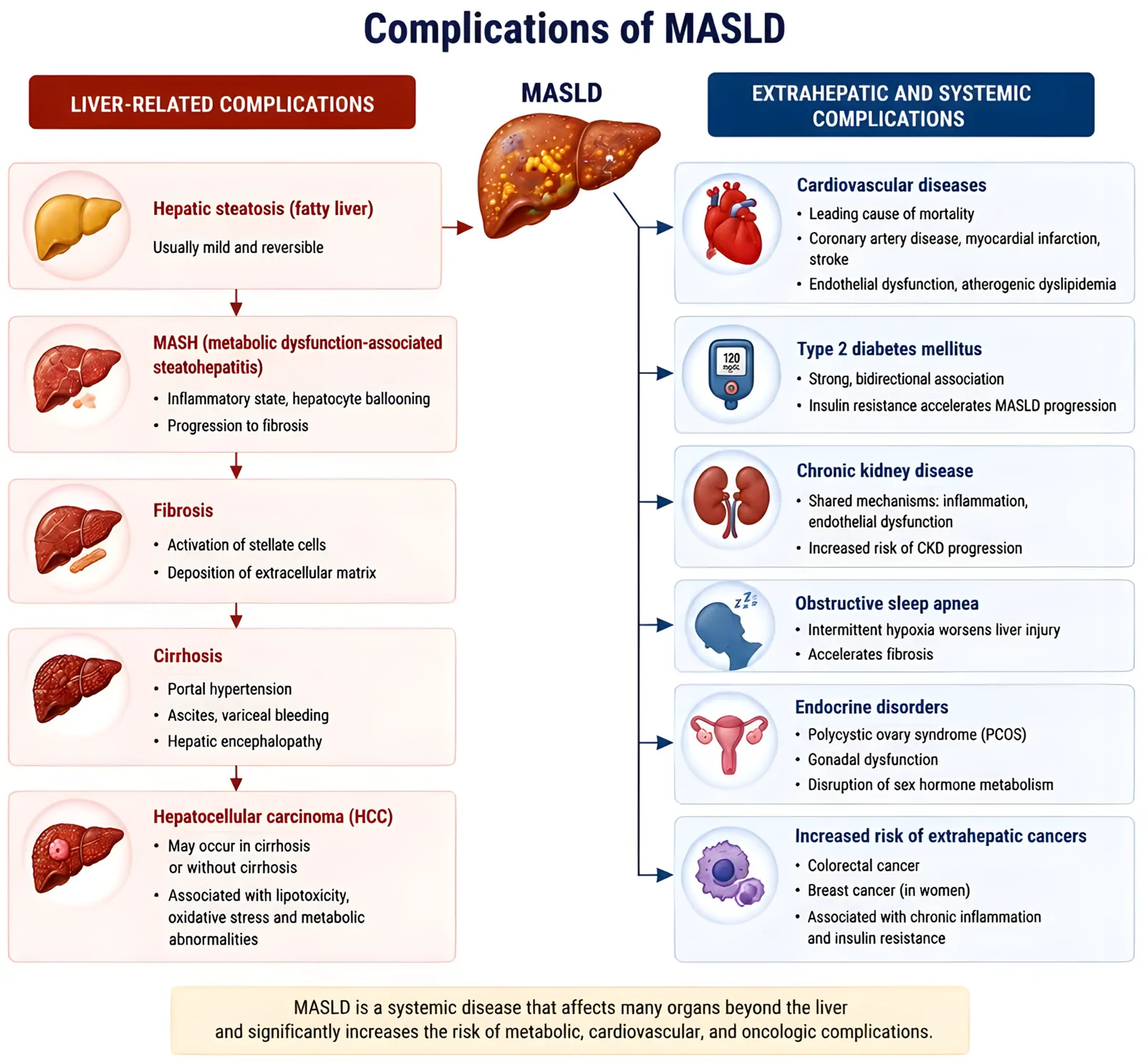
Complications of metabolic dysfunction-associated steatotic liver disease (MASLD).

The disease forms a dynamic spectrum in which isolated steatosis progresses to MASH in 10–20% of cases, triggering ballooning degeneration, lipotoxicity, and parenchymal injury (Figure 1) [11,15]. Persistent inflammation activates hepatic stellate cells, leading to extracellular matrix deposition, with the stage of histological fibrosis serving as the primary prognostic factor for survival [15,19]. Progressive fibrosis inevitably leads to cirrhosis, structural distortion of the organ, and complications of portal hypertension [15]. Of particular note is the hepatocellular carcinoma paradox, which complicates clinical surveillance because lipotoxicity and systemic metabolic dysregulation frequently induce oncogenesis in a non-cirrhotic liver, unlike in HCV infection or alcoholic liver disease [20,21].

The change in nomenclature reflects the multisystem nature of the disorder [13]. The steatotic liver acts as an active endocrine organ, releasing pro-inflammatory cytokines, hepatokines, and procoagulant factors [22]. It exhibits a strong, bidirectional relationship with T2DM, driven by peripheral insulin resistance, where both conditions mutually accelerate each other’s progression [23]. Furthermore, the steatotic environment exacerbates atherosclerosis, significantly increasing the risk of cardiovascular events through endothelial dysfunction and dyslipidemia [12,14,19]. For this reason, the disease is widely recognized as the hepatic component of metabolic syndrome [22].

Ultimately, chronic low-grade inflammation and insulin resistance link MASLD to a range of extrahepatic conditions. It represents an independent risk factor for chronic kidney disease [19] and shows a strong association with obstructive sleep apnea, in which hypoxic episodes accelerate fibrosis [22]. It is also linked to polycystic ovary syndrome and gonadal disorders [22], as well as a higher incidence of extrahepatic malignancies, including notably colorectal cancer and breast cancer in women, emphasizing the need for interdisciplinary patient surveillance [17,22].

## 3. Etiopathogenesis

**Figure 2:**
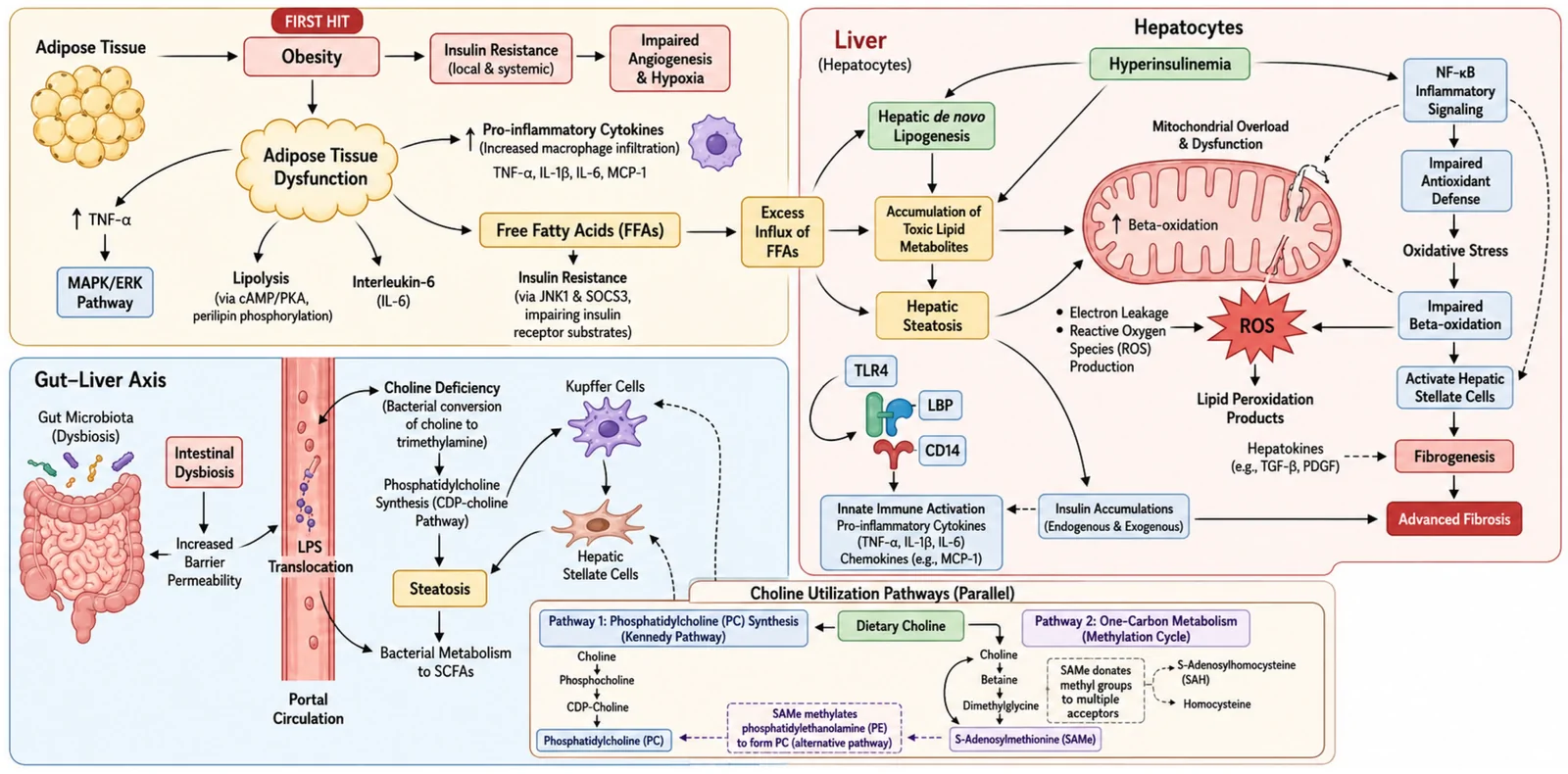
Etiopathogenesis of metabolic dysfunction-associated steatotic liver disease (MASLD).

MASLD development is complex and multifactorial. The first pathogenetic model of metabolic dysfunction-associated steatotic liver disease was the “two-hit hypothesis,” in which the first hit involved lipid accumulation within hepatocytes, while the second hit comprised factors triggering inflammation and hepatocyte injury, ultimately leading to liver fibrosis [24]. Over time, this model was replaced by the “multiple-hit hypothesis,” in which coexisting metabolic, inflammatory, genetic, and environmental mechanisms interact and potentiate one another, driving disease progression [25]. Pathogenesis typically begins with obesity-induced adipose tissue dysfunction and early insulin resistance, which together drive impaired angiogenesis, local hypoxia, worsening systemic insulin resistance, and the activation of fibrogenic pathways (Figure 2). Hyperinsulinemia promotes the release of free fatty acids and activates nuclear factor kappa B inflammatory signalling in the liver, resulting in the accumulation of toxic lipid metabolites within hepatocytes. Excess free fatty acids further exacerbate insulin resistance in skeletal muscle and promote hepatic de novo lipogenesis. Circulating free fatty acids are an independent prognostic factor for advanced fibrosis in metabolic dysfunction-associated steatotic liver disease [26,27,28]. Progressive adipose tissue dysfunction also promotes macrophage infiltration and increased production of pro-inflammatory cytokines, particularly tumour necrosis factor alpha [27]. Tumour necrosis factor alpha activates the mitogen-activated protein kinase/extracellular signal-regulated kinase pathway and enhances lipolysis through the cyclic adenosine monophosphate/protein kinase A system, leading to perilipin phosphorylation and increased release of free fatty acids. This process contributes to hepatic steatosis and the progression of metabolic dysfunction-associated steatotic liver disease [29]. Visceral adipocytes also secrete interleukin-6, which induces insulin resistance through activation of c-Jun N-terminal kinase 1 and suppressor of cytokine 3, impairing insulin signalling and accelerating degradation of insulin receptor substrates [30]. Excessive influx of free fatty acids into hepatocytes results in mitochondrial overload and dysfunction. Initially, beta-oxidation is increased, leading to enhanced electron leakage within the respiratory chain and increased production of reactive oxygen species. The resulting oxidative stress damages the mitochondrial membranes and cellular structures of hepatocytes. In later stages, impaired beta-oxidation leads to further lipid accumulation, exacerbation of inflammation, and worsening insulin resistance. In addition, lipid peroxidation products activate hepatic stellate cells, initiating the fibrotic process [26,31,32]. An important component of the pathogenesis of MASLD is the gut–liver axis. Intestinal dysbiosis and increased intestinal barrier permeability facilitate the translocation of lipopolysaccharide into the portal circulation. Lipopolysaccharide activates Toll-like receptor 4, which is expressed on Kupffer cells, hepatocytes, and hepatic stellate cells, inducing an inflammatory response involving cluster of differentiation 14 and lipopolysaccharide-binding protein [33,34]. The gut microbiota also influences key metabolic pathways associated with hepatocyte function. Choline deficiency, partly resulting from its bacterial conversion into methylamines, leads to reduced phosphatidylcholine synthesis and disturbances in the S-adenosylmethionine pathway, thereby promoting the development of hepatic steatosis [33,35]. Additionally, bacterial metabolism of phenylalanine into phenylacetic acid has been associated with the development of MASH, and phenylacetic acid itself may serve as a biomarker with high predictive value for this disease phenotype [33].

## 4. Diagnostics and Assessment of Disease Severity

The initial biochemical markers that assess of liver injury is based on the measurement of alanine aminotransferase (ALT), aspartate aminotransferase (AST), and gamma-glutamyl transferase (GGT). In patients with MASLD, aminotransferase activity may be mildly to moderately elevated; however, in some cases, it remains within the normal range [36]. ALT, predominantly localized in the cytoplasm of hepatocytes, is a sensitive marker of hepatocellular injury and hepatic steatosis, whereas a relative predominance of AST, present in both the cytoplasm and mitochondria, may indicate more advanced liver damage. GGT activity often increases with the progression of MASLD and is associated with oxidative stress. This enzyme is involved in glutathione metabolism, and its increased expression may serve as a marker of oxidative stress in MASLD [37].

Since isolated liver enzymes lack sufficient specificity to accurately assess the stage of liver fibrosis, several non-invasive algorithms have been developed that combine these enzymes with various clinical variables, as well as scoring systems based on additional clinical and laboratory parameters.

## 5. Non-Invasive Fibrosis Assessment Scores

The Fibrosis-4 (FIB-4) index is a widely used, non-invasive scoring system for assessing the degree of liver fibrosis, based on patient age, ALT, AST, and platelet count [37]. It is recommended as a first-line screening tool to exclude advanced fibrosis, particularly in primary care settings. A FIB-4 value < 1.30 provides a high negative predictive value, effectively identifying low-risk patients who do not require further specialist evaluation [4,5], whereas a value > 2.67 indicates a high risk of advanced fibrosis and warrants further diagnostic assessment [35]. Due to the significant impact of age on the score, adjusted cut-off values (e.g., >2.0) are recommended for patients older than 65 years to reduce false-positive results [5].

The NAFLD Fibrosis Score (NFS) was developed to assess the risk of advanced fibrosis in patients with MASLD. It includes six parameters: age, body mass index, impaired fasting glucose or the presence of diabetes, the ALT/AST ratio, platelet count, and serum albumin level. Like the FIB-4 index, it allows for the stratification of patients into risk groups. A value < −1.455 excludes advanced fibrosis, whereas a score > 0.675 indicates a high probability of its presence. Despite its high clinical usefulness, the results may be influenced by extreme body mass index values and patient age, and therefore require cautious interpretation [5].

The aspartate aminotransferase to platelet ratio index (APRI) is a non-invasive marker of liver fibrosis based on the relationship between elevated AST activity and decreased platelet count. It is calculated using the following formula [38]:

(AST level/upper limit of normal for AST)/platelet count (10^9^/L) × 100

This index demonstrates acceptable diagnostic utility in assessing the degree of fibrosis in the later stages of MASLD. This is mainly due to the typically normal platelet count and only mild to moderate elevations in AST activity observed in the early stages of fibrosis in this condition [38,39].

The AST to ALT ratio (De Ritis ratio) is an easily accessible marker associated with hepatic steatosis. In the early stages of MASLD, ALT activity is typically higher than AST, resulting in a low De Ritis ratio (AST/ALT < 1), which may be related to increased insulin resistance, oxidative stress, and lipotoxicity leading to hepatocellular injury. As the disease progresses to more advanced stages, such as advanced fibrosis or cirrhosis, this ratio tends to increase and reverse (AST/ALT > 1), making it a useful indicator of disease progression [36].

## 6. Liver Function and Prognostic Scoring Systems

The Child–Pugh classification was developed in 1973 as a scoring system to assess prognosis in patients with liver disease, originally in the context of bleeding from esophageal varices. It is currently widely used as a bedside tool for evaluating the degree of liver failure and prognosis in cirrhosis. However, in the context of liver transplantation eligibility, it has largely been replaced by the Model for End-Stage Liver Disease (MELD). The scale includes five parameters: ascites, hepatic encephalopathy, nutritional status, total bilirubin, serum albumin, and prothrombin time or the international normalized ratio. Each parameter is scored from 1 to 3 points, allowing patients to be classified into three severity classes: A (5–6 points), B (7–9 points), and C (10–15 points), corresponding to mild, moderate, and severe liver cirrhosis, respectively [40,41,42].

MELD is a scoring system based on serum creatinine, bilirubin, and the international normalized ratio. It was originally developed to assess prognosis in patients undergoing transjugular intrahepatic portosystemic shunt procedures and is currently the primary tool for organ allocation in patients awaiting liver transplantation due to its ability to predict short-term mortality. A modification of this score, the MELD incorporating sodium, additionally includes serum sodium concentration in the scoring criteria. This model is associated with significantly lower mortality (hazard ratio: 0.74) and a higher probability of transplantation (hazard ratio: 1.22). This is because hyponatremia (below 126 milliequivalents per liter) reflects the severity of portal hypertension and is associated with complications such as ascites and hepatorenal syndrome, which may negatively affect liver transplant outcomes [41,43].

## 7. Conventional Imaging Techniques

Conventional B-mode ultrasonography is a primary, non-invasive diagnostic method used in the diagnosis of MASLD. Although it allows for the assessment of liver steatosis using a four-grade scale, in clinical practice, it is typically limited to a qualitative assessment (presence or absence of steatosis). This method is characterized by limited sensitivity in the early stages of the disease and in patients with obesity, with the highest diagnostic efficacy observed in cases with steatosis ≥ 12.5%. Consequently, it does not reliably allow for the exclusion of fibrosis or MASH [8,44,45,46,47].

Computed tomography can serve as a supplementary method; it is a rapid and widely available imaging technique used to assess liver steatosis, in which the degree of steatosis correlates with a decrease in attenuation values. Non-contrast computed tomography enables the detection of mild to moderate steatosis, while contrast-enhanced computed tomography additionally allows for the assessment of liver perfusion and function, which is useful in monitoring complications of MASLD [8,48,49].

An advancement of classical computed tomography is dual-energy computed tomography, a technique with higher diagnostic precision that enables the simultaneous acquisition of images at two different X-ray energy levels. It allows for a more accurate quantitative assessment of hepatic fat content and other tissue components, thereby improving the detection of early stages of MASLD and the assessment of complication risks [48,50].

## 8. Multiparametric Imaging

Vibration-controlled transient elastography (VCTE) is the most used technique in clinical practice, enabling the assessment of liver stiffness and the measurement of controlled attenuation parameters. In combination with AST levels, these parameters are used to calculate the FAST score, which is a useful predictor of MASH and may partially replace liver biopsy. This method also has prognostic value in assessing the risk of liver cirrhosis, HCC, and the need for liver transplantation; however, its accuracy may be limited by obesity, ascites, and operator-dependent factors [51,52,53].

An alternative to transient elastography is acoustic radiation force impulse (ARFI) elastography, which demonstrates comparable diagnostic accuracy, particularly in advanced fibrosis. At the same time, it is less affected by confounding factors and can be easily integrated into standard ultrasound systems. Nevertheless, VCTE remains the most widely used technique in clinical practice [44,54].

The highest accuracy in non-invasive assessment of liver fibrosis is achieved with magnetic resonance elastography (MRE), which is considered one of the most precise methods for evaluating liver fibrosis, particularly in its early stages. This technique assesses the propagation of mechanical waves using magnetic resonance imaging and demonstrates higher diagnostic accuracy than VCTE in obese patients. However, its clinical use is limited by its high cost and susceptibility to artefacts in patients with iron overload [53,55].

In addition to fibrosis assessment, accurate quantitative evaluation of hepatic steatosis is an essential component of MASLD diagnostics. Proton density fat fraction magnetic resonance imaging (MRI-PDFF) is currently considered the most precise non-invasive method for this purpose. Proton density fat fraction correlates with histological changes and treatment response, often before clinical improvement becomes apparent, making MRI-PDFF a valuable tool in research on MASLD and in therapeutic trials. Its results have also been shown to correlate with the risk of developing MASH, steatotic liver disease progression, and HCC [56,57,58].

## 9. Microscopic Features of Hepatic Steatosis

In MASLD, the dominant type of hepatic steatosis is macrovesicular steatosis, characterized by the presence of one or several large lipid droplets that displace the hepatocyte nucleus to the periphery. This lipid accumulation typically begins in zone 3 of the liver lobule. Much less common is the microvesicular variant, characterized by numerous small lipid droplets in the cytoplasm with the nucleus remaining in a central position [59,60]. As these changes progress, hepatocyte ballooning becomes a key marker of disease advancement. These are enlarged cells with rarefied, reticulated cytoplasm, reflecting cellular injury and the onset of fibrogenic activity [59,60]. This process is accompanied by an inflammatory response, manifesting as mild to moderate lobular inflammatory infiltrates consisting of lymphocytes, macrophages (Kupffer cells), neutrophils, and eosinophils. Characteristic features in this area include lipogranuloma and satellitosis, the latter referring to the accumulation of inflammatory cells around damaged hepatocytes containing Mallory-Denk bodies [60]. These bodies are cytoplasmic inclusions composed of damaged intermediate cytokeratin filaments, ubiquitin, and p62 protein, which result from disruption of cytoskeletal homeostasis [59,61]. Histological assessment of the complex MASLD pathology relies on haematoxylin and eosin (HE) staining and specialized staining and immunohistochemical techniques. Masson’s trichrome staining allows for the evaluation of fibrosis, while Sirius red staining enables precise quantitative assessment of collagen deposition, particularly in the early stages of the disease. Gordon-Sweet staining is used to evaluate the reticulin network structure and the degree of architectural distortion of the liver parenchyma [62,63,64,65]. This diagnostic approach is complemented by immunohistochemical methods, including staining for cytokeratin 8 and 18, ubiquitin, and p62 protein, which support the identification of Mallory-Denk bodies and the assessment of hepatocyte injury. Furthermore, cytokeratin 18 fragments (e.g., M30) serve as circulating biomarkers for evaluating apoptotic-inflammatory activity and the severity of metabolic dysfunction-associated steatohepatitis [59,60,66,67].

## 10. Fibrotic Changes (Stages 0–4 According to the Kleiner/NASH-CRN Scale)

Liver fibrosis is the most important prognostic factor in MASLD, strongly correlating with the risk of complications and mortality. The process typically begins in zone 3 of the hepatic lobule, with perisinusoidal and pericellular collagen deposition, forming a characteristic “chicken wire” pattern. Assessment of fibrosis stage is based on the NASH-CRN (Kleiner) scoring system:

- F0—No fibrosis;
- F1—Zone 3 (perisinusoidal) and/or portal/periportal fibrosis:
  o F1a—Mild pericellular fibrosis in zone 3 (visible only with special connective tissue staining);
  o F1b—Moderate pericellular fibrosis in zone 3 (visible with routine H&E staining);
  o F1c—Portal/periportal fibrosis without pericellular fibrosis;
- F2—Combined perisinusoidal and portal fibrosis;
- F3—Bridging fibrosis;
- F4—Cirrhosis.

Early F1 stages may include different patterns of fibrosis, including changes visible only in connective tissue stains or hematoxylin and eosin staining, as well as isolated portal fibrosis typical of paediatric metabolic dysfunction-associated steatotic liver disease [59,60].

For clinical and histopathological reasons, the NASH-CRN system must be distinguished from the five-stage METAVIR scale (F0–F4). The METAVIR scale was originally developed to evaluate liver fibrosis in viral hepatitis and is based on assessing portal tract expansion and the formation of portal-portal septa. Unlike NASH-CRN, however, it does not account for perisinusoidal fibrosis in zone 3 of the hepatic lobule [59,60,68,69].

## 11. Differentiation from Non-Alcoholic Steatohepatitis

The main purpose of liver biopsy is to differentiate simple steatosis from MASH. Simple steatosis is diagnosed in the presence of lipid accumulation in hepatocytes without signs of active liver cell injury, whereas the diagnosis of MASH requires the simultaneous presence of steatosis, lobular inflammation, and hepatocellular ballooning [59]. To standardize histopathological assessment, the NFS is used, which sums the severity of steatosis (0–3), lobular inflammation (0–3), and hepatocellular ballooning (0–2). This score is primarily used to assess disease activity and monitor histological changes rather than serve as an independent diagnostic criterion for MASH [59,70]. Despite its high clinical value, liver biopsy has limitations that preclude its use as a screening tool in large populations [71]. Percutaneous liver biopsy is associated with patient discomfort and a risk of complications, most commonly pain, while in 1–3% of cases, post-procedural bleeding requiring hospitalization may occur [72]. A standard biopsy sample represents only a very small portion of the liver, and the heterogeneity of inflammatory and fibrotic changes increases the risk of sampling error, even between different liver lobes [73]. In addition, conventional semi-quantitative histological assessment based on human interpretation is characterized by significant intra- and inter-observer variability [74]. Work is ongoing to develop new approaches to minimize these limitations. To reduce sampling error, endoscopic ultrasound-guided liver biopsy has been introduced, allowing tissue collection from both liver lobes in a single procedure [72]. Digital pathology and artificial intelligence algorithms, such as advanced fluorescence imaging, enable objective, automated, and fully quantitative assessment of collagen structure and hepatic steatosis [75].

## 12. Non-Pharmacological Management

Weight loss is a key component of the non-pharmacological treatment of MASLD, regardless of the stage of the disease. Clinical guidelines indicate that a daily caloric deficit of 500–1000 kcal in patients with MASLD leads to weight reduction accompanied by a decrease in hepatic steatosis, regardless of macronutrient composition. For most patients, the therapeutic goal is a 7–10% reduction in body weight [6]. Dietary measures are effectively supported by regular physical activity, which constitutes another key pillar of therapy. Studies have shown that 3–5 sessions of moderate or vigorous exercise per week reduce the risk of developing MASLD. Furthermore, regular physical activity favorably modifies the lipid profile, lowers blood pressure, and contributes to weight loss [76]. A 12-month intensive lifestyle intervention, including dietary modification and increased physical activity in patients with type 2 diabetes and a body mass index above 25 kg/m^2^, resulted in an average weight loss of 8%, together with a significant reduction in liver steatosis and the prevalence of MASLD compared with the control group [77]. The preferred dietary pattern for patients with MASLD is the Mediterranean diet, which, due to its high content of monounsaturated fatty acids, reduces cardiovascular risk, improves insulin sensitivity, and decreases hepatic steatosis. Studies conducted in patients with insulin resistance and MASLD have shown that the Mediterranean diet is associated with only modest weight loss and does not significantly change body mass index or waist circumference compared with the low-fat, high-carbohydrate diet used in control groups. However, individuals following the Mediterranean diet demonstrated significant improvement in insulin sensitivity, lower serum insulin levels, and reduced liver fat content [78]. In more advanced cases, bariatric surgery is an important therapeutic option for obese patients with MASLD or MASH. Compared with baseline values, a significant improvement in hepatic steatosis was observed both one year and five years after surgery, without worsening of procedure-related fibrosis [9].

## 13. Pharmacological Management

Pharmacological treatment of MASLD includes agents aimed at improving liver function and systemic metabolic homeostasis, particularly glucose and lipid metabolism. The choice of therapy depends mainly on the presence of type 2 diabetes, obesity, and cardiovascular risk factors.

### 13.1. THRβ Agonists

Resmetirom is a selective thyroid hormone receptor-beta (THR-β) agonist with preferential hepatic activity. In March 2024, it was granted accelerated approval from the U.S. Food and Drug Administration as the first targeted drug for the treatment of MASH in adults with F2–F3 fibrosis but without liver cirrhosis [79]. In the pathogenesis of MASLD/MASH, disruptions of the thyroid hormone axis and THR-β-dependent pathway activity are observed, which may contribute to impaired lipid metabolism and mitochondrial dysfunction in hepatocytes [80]. Because THR-β is the dominant thyroid hormone receptor isoform in the liver, its selective activation by resmetirom enhances fatty acid β-oxidation, increases lipid metabolism, and promotes mitochondrial biogenesis, thereby reducing lipid accumulation and associated lipotoxicity phenomena [80,81]. Selectivity toward THR-β simultaneously limits the impact on THR-α, whose activation is responsible for part of the systemic effects of excess thyroid hormones. However, this does not imply a complete absence of adverse effects; in clinical trials, gastrointestinal disorders and pruritus were among the most frequently observed [81]. The efficacy of resmetirom was evaluated, among other drugs, in the randomized phase III MAESTRO-NASH trial involving 966 patients with biopsy-confirmed MASH and F1–F3 fibrosis, with the primary histological analysis population comprising patients with more advanced fibrosis. After 52 weeks of treatment, MASH resolution without worsening of fibrosis was achieved in 25.9% of patients receiving 80 mg and 29.9% of those receiving 100 mg of resmetirom, compared to 9.7% in the placebo group. Improvement in fibrosis by at least one stage without worsening of MASH was achieved in 24.2% and 25.9% of patients in the resmetirom groups, respectively, versus 14.2% of patients receiving placebo [82]. In addition to its effect on histological features of MASH, resmetirom exhibits metabolic effects, including a reduction in atherogenic lipid fractions, as well as favorable changes in selected liver parameters and thyroid hormone axis markers [83].

### 13.2. PPAR-γ Agonists

In patients with type 2 diabetes co-occurring with MASLD or MASH, peroxisome proliferator-activated receptor γ (PPAR-γ) agonists, including pioglitazone, demonstrate favorable therapeutic effects. By activating these receptors, pioglitazone improves peripheral insulin sensitivity and inhibits hepatic glucose production. Furthermore, pioglitazone possesses potent anti-inflammatory properties by suppressing the expression of pro-inflammatory mediators in hepatocytes, as well as limiting pyroptosis pathways and NLRP3 inflammasome formation. The drug’s antifibrotic action is based on inhibiting the activation of hepatic stellate cells and TGF-beta signaling, which results in reduced procollagen expression and modified extracellular matrix metalloproteinase activity [84]. The outcome of these mechanisms is a reduction in liver enzyme activity and an improvement in glycemic control, manifested by reduced fasting glucose levels and HbA1c, with no significant effect on the lipid profile. In MASLD patients without T2DM, the drug also effectively reduces steatosis and inflammation severity, although weight gain is reported more frequently in this group compared to diabetic patients [10,85]. Long-term treatment with pioglitazone is associated with a beneficial impact on the course of MASH, leading to a reduction in steatosis, hepatocyte ballooning, and the degree of fibrosis [86]. Meta-analyses indicate an increased likelihood of complete MASH resolution and regression of advanced fibrosis, regardless of the patient’s metabolic status. Our own study results confirm these observations: long-term therapy (4–10 years) led to a statistically significant improvement in enzymatic parameters and non-invasive markers, such as CAP and the FAST™ score, documenting the drug’s efficacy in halting disease progression [87]. Despite proven clinical benefits, the use of pioglitazone carries a risk of adverse effects. The most common include fluid retention, a potential increase in the incidence of bladder cancer, and weight gain, which averaged 4.6 kg in long-term studies (96 months) [10].

### 13.3. GLP-1R Agonists

Glucagon-like peptide-1 receptor agonists (GLP-1RAs) represent an important class of drugs used in the treatment of metabolic disorders associated with MASLD, particularly T2DM and obesity. By stimulating glucose-dependent insulin secretion, suppressing glucagon secretion, delaying gastric emptying, and increasing satiety, they lead to improved glycemic control and weight reduction. Selected agents from this class also demonstrate a favorable effect on cardiovascular risk in patients with metabolic disorders [88,89,90,91]. In the context of MASLD/MASH, the efficacy of GLP-1RAs is primarily linked to weight loss, improved insulin sensitivity, and a reduction in hepatic steatosis. Clinical trials have also shown an increase in the proportion of patients achieving MASH resolution without worsening of fibrosis. However, data regarding the direct impact of GLP-1RAs on fibrosis regression have previously remained less definitive and depended on the specific drug used and the study population. Among the representatives of this class, semaglutide has gained particular importance. Results from clinical trials, including the ESSENCE trial, demonstrated its efficacy in achieving MASH resolution as well as a favorable effect on metabolic parameters in patients with MASH and stage F2–F3 fibrosis [92]. In 2025, semaglutide was approved by the FDA for the treatment of MASH in adult patients with moderate to advanced fibrosis (F2–F3), marking a significant step forward in the pharmacotherapy of this disease [93].

### 13.4. SGLT-2 Inhibitors

Sodium-glucose cotransporter 2 (SGLT-2) inhibitors lower blood glucose levels by inhibiting renal glucose reabsorption in the proximal tubules. They improve metabolic control by reducing HbA1c levels, lowering insulin requirements, and promoting weight loss, while simultaneously exerting cardioprotective effects [94]. Empagliflozin increases nitric oxide release from blood vessels, which facilitates vasodilation and exhibits anti-atherosclerotic properties [95]. Furthermore, 48-week therapy with dapagliflozin led to significant improvement in MASH without worsening of fibrosis in 53% of patients (compared to 30% in the placebo group), also contributing to the resolution of steatohepatitis and regression of fibrosis. Additionally, treatment with the drug resulted in a decrease in liver enzyme levels, a reduction in hepatic steatosis and liver stiffness, and improvements in metabolic parameters such as body weight, blood glucose, and blood pressure [96].

### 13.5. Vitamin E

Vitamin E, which exhibits antioxidant properties, is also used in treatment and yields beneficial effects in patients with MASLD. At doses of 400–800 IU (268–536 mg), it reduces alanine and aspartate aminotransferase activity and limits hepatic steatosis, inflammation, and hepatocellular ballooning, without exerting a significant effect on liver fibrosis [9,10]. However, given the lack of conclusive evidence from large phase III trials regarding histological improvement, as well as potential long-term risks, it is not recommended as a targeted therapy for MASH [8].

### 13.6. Statins

According to guidelines, statin therapy is recommended for patients with MASLD and dyslipidemia, as these drugs contribute to lowering low-density lipoprotein (LDL) cholesterol levels, improving lipid profiles, and reducing the incidence of major adverse cardiovascular events [97,98]. Statins act as inhibitors of 3-hydroxy-3-methylglutaryl-CoA reductase, the enzyme responsible for converting HMG-CoA to mevalonate. A characteristic feature of these agents is their pleiotropic multi-target activity, which regulates lipid metabolism in terms of lipogenesis-lowering cholesterol and triglyceride concentrations and enhancing the activity of the antioxidant enzyme PON1, thereby mitigating oxidative stress [99,100]. Furthermore, statins demonstrate potent anti-inflammatory properties by suppressing the expression of key pro-inflammatory cytokines, such as TNF-alpha and IL-6. Their antifibrotic activity relies on blocking the activation of hepatic stellate cells and suppressing TGF-beta-dependent profibrotic signaling pathways. These drugs also improve the function of hepatic sinusoidal endothelial cells by enhancing nitric oxide signaling [101]. Moreover, in the realm of anticarcinogenesis, statins exhibit a potential protective effect against the development of HCC, reducing the risk by 53 percent through the downregulation of inflammatory markers and growth factor expression [102,103]. Nevertheless, statin administration in MASLD raises concerns regarding elevations in liver enzyme levels, although according to guidelines, these changes are typically mild, transient, and clinically insignificant. In elderly patients, the risk of adverse effects is heightened due to physiological changes and polypharmacy. The primary complications of concern in this population include myopathy, rhabdomyolysis, and elevated liver enzymes [97].

### 13.7. Metformin

Metformin is a widely used antidiabetic drug that modulates glucose and lipid metabolism through the activation of the AMP-activated protein kinase pathway and the inhibition of hepatic glucose production. This compound contributes to a reduction in free fatty acid levels and the limitation of harmful lipogenic processes. Furthermore, it demonstrates proven hepatoprotective effects, consisting of mitigation of cellular stress, suppression of inflammation, and inhibition of liver fibrosis processes [104]. Although metformin pharmacotherapy in patients with MASLD promotes improved insulin sensitivity and the normalization of biochemical markers, weight loss is considered the key driver inducing these favorable changes [105]. Study results indicate that both metformin monotherapy and its combination therapy with the Ganwei preparation in patients with MASLD lead to a significant reduction in body weight, glycated hemoglobin levels, and the activity of AST and ALT after six months. Moreover, combination therapy provides additional metabolic benefits in the form of lowered total cholesterol and LDL-fraction levels, as well as improved renal function, manifested by an increase in estimated glomerular filtration rate and a decrease in creatinine levels [106]. However, the use of metformin in the adolescent population with MASLD remains controversial due to therapeutic limitations and potential adverse effects, with patient response to treatment showing high variability driven by individual enzymatic profiles. The standard dose of the drug reduces hepatic glucose production by an average of 19%, with approximately 15% of pediatric patients showing a lack of sensitivity to therapy. In this patient group, elevated levels of lactate or insulin during an oral glucose tolerance test can be classified as prognostic markers of good therapeutic response [107].

## 14. Surgical Management

Due to the rapidly increasing prevalence and severity of MASLD, it has become the second most common indication for liver transplantation in the United States. Transplant candidates face numerous challenges, such as morbid obesity (body mass index ≥ 40 kg/m^2^), which is often considered a contraindication. Patients with MASLD have an increased risk of removal from the transplant waiting list because of comorbidities, advanced age, impaired renal function, and lower MELD scores. Extensive portal vein thrombosis, which frequently occurs in cirrhosis associated with MASLD, may also lead to removal from the waiting list because of the high risk of perioperative morbidity and mortality. Overall, liver transplantation outcomes are very good; however, close monitoring is required during the first six months after transplantation because of the increased risk of mortality during this period [108].

## 15. Investigational Drugs

### 15.1. Galectin-3 Inhibitors

Galectin-3 is a protein with a complex role in the pathogenesis of MASLD that depends on both cell type and disease stage. Experimental studies suggest that, in the early stages of the disease, galectin-3 may be involved in the regulation of lipid metabolism and the cellular stress response, whereas with disease progression, its increased expression in activated hepatic stellate cells and immune cells may contribute to chronic inflammation and the activation of fibrogenic processes. The complexity of these interactions indicates that the effects of galectin-3 may vary depending on the stage of disease and cellular context, which should be taken into account when evaluating its potential as a therapeutic target [109]. Given the involvement of galectin-3 in inflammatory and fibrotic processes, its inhibition is currently being investigated as a potential therapeutic strategy for MASLD/MASH. One of the most extensively studied galectin-3 inhibitors is belapectin. In the NAVIGATE trial, its effect on the development of clinically significant esophageal varices was evaluated in patients with MASH-related cirrhosis and portal hypertension. The results indicated a reduced risk of developing new esophageal varices while demonstrating a favorable safety profile [110]. These findings support further investigation of galectin-3 inhibition, particularly with regard to its potential antifibrotic effects. Another compound in this class is selvigaltin, an oral small-molecule galectin-3 inhibitor. Preclinical studies have demonstrated its potential anti-inflammatory and antifibrotic effects, including attenuation of the activation of cells involved in MASH progression and a reduction in the severity of histopathological changes. Given the relatively early stage of its clinical development, the efficacy of selvigaltin in improving hepatic fibrosis and histological activity of MASH requires confirmation in larger, randomized clinical trials [111].

### 15.2. FGF21 Analogs

Another promising class of therapies comprises fibroblast growth factor 21 (FGF21) analogs. FGF21 is a peptide hormone produced mainly in the liver and is involved in the regulation of lipid and glucose metabolism. FGF21 increases fatty acid oxidation, influences lipid metabolism, and improves insulin sensitivity, while its activity is also associated with potential anti-inflammatory and antifibrotic effects. Therefore, FGF21 analogues are being extensively investigated as potential therapies targeting metabolic disturbances, hepatic steatosis, and liver fibrosis simultaneously [112,113]. One of the most clinically advanced FGF21 analogues is efruxifermin, a long-acting FGF21 analog administered subcutaneously once weekly. In the randomized phase 2b HARMONY trial, which included patients with biopsy-confirmed MASH and fibrosis stage F2–F3, the efficacy and safety of efruxifermin were assessed compared with placebo over a 96-week period. Treatment resulted in significant improvements in parameters related to hepatic steatosis and fibrosis, while some patients also demonstrated simultaneous improvement in histological MASH activity and fibrosis stage. These findings support the therapeutic potential of the FGF21 axis; however, further validation in phase 3 trials and with regard to long-term clinical outcomes is required [114]. Another promising member of this class is pegozafermin, a long-acting, PEGylated FGF21 analog. In phase 2b clinical trials, it demonstrated beneficial effects on parameters of MASH activity and liver fibrosis, supporting the further clinical development of this compound [115].

### 15.3. Triple PPAR Agonists

Pioglitazone, a well-known conventional pharmacotherapy, has confirmed the efficacy of PPAR pathway activation in reducing hepatic steatosis; however, its clinical application is limited by its selective targeting of only the PPAR-γ. Lanifibranor represents an evolution of this strategy, which is in clinical trials. As a pan-PPAR agonist, unlike pioglitazone, it activates all three PPAR isoforms (PPAR-α, PPAR-γ, and PPAR-β/δ). This comprehensive approach targets key stages of the MASH disease cascade. By activating the PPAR-α isoform, the drug enhances fatty acid β-oxidation, preventing excessive lipid accumulation in hepatocytes. Concurrently, the δ and γ components attenuate the inflammatory response and inhibit the transdifferentiation of hepatic stellate cells into myofibroblasts, which are responsible for fibrogenesis. Furthermore, PPAR-γ stimulation reduces peripheral insulin resistance and improves adipose tissue function, alleviating the systemic metabolic burden on the liver [116]. In a phase 2b trial in patients with active MASH, lanifibranor induced significant histological improvement, leading to both MASH resolution and fibrosis regression without disease progression. The drug’s mechanism also translated into broader metabolic benefits, including improved glycemic control in diabetic patients, increased High-Density Lipoprotein (HDL) cholesterol levels, reduced triglycerides, and elevated adiponectin concentrations. Despite a minor weight gain and peripheral edema, the multifaceted mode of action makes lanifibranor a promising candidate currently being evaluated in phase 3 trials [117].

### 15.4. Dual PPAR Agonists

In addition to pan-PPAR agonists, dual agonists such as saroglitazar are also undergoing clinical evaluation. As a dual PPAR-α/γ agonist, saroglitazar alleviates hypertriglyceridemia and mixed dyslipidemia via PPAR-α activation while enhancing glycemic control through PPAR-γ-dependent pathways [118]. When it was administered for 24 weeks to patients with MASLD, it significantly reduced hepatic steatosis and liver stiffness, as demonstrated by lower Controlled Attenuation Parameter and Liver Stiffness Measurement scores. It also exhibited potent metabolic efficacy, marked by a nearly 50% reduction in triglyceride levels and improved glycemic control with an HbA1c reduction of 0.63%. Crucially, these hepatoprotective effects and improvements in insulin sensitivity were achieved without requiring weight loss or strict dietary restrictions [119]. Another molecule evaluated in this class was elafibranor, a dual PPAR-α/δ agonist. However, despite promising post-hoc analysis results from phase 2 trials showing NASH resolution without worsening of fibrosis, it failed to meet its primary histological endpoint in a pivotal phase 3 trial, leading to the termination of its development for this indication [120].

### 15.5. Selective PPAR-α Modulator

Another class of agents targeting PPAR includes selective PPAR-α modulators (SPPARMα), with pemafibrate being the most clinically advanced representative. By stimulating lipoprotein lipase activity, pemafibrate reduces plasma levels of triglycerides and VLDL particles. In the PROMINENT trial, it failed to demonstrate a reduction in major adverse cardiovascular events, limiting its utility in cardiovascular prevention [121]. Nevertheless, pemafibrate remains under investigation for its therapeutic potential in MASLD among patients with concurrent hypertriglyceridemia, primary biliary cholangitis, and dyslipidemia [113].

### 15.6. Dual GLP-1R/GIPR Agonists

An evolution of GLP-1R agonist therapy is the class of dual Glucagon-Like Peptide-1 Receptor and Glucose-Dependent Insulinotropic Polypeptide Receptor (GLP-1R/GIPR) agonists, whose mechanism of action combines the metabolic effects of both incretin pathways. GLP-1R exert direct hepatoprotective effects via the activation of AMPK kinase and the PI3K/Akt pathway, which inhibits lipogenesis, enhances fatty acid beta-oxidation, and suppresses inflammation and fibrosis in the liver. Conversely, GIPR agonists act primarily in adipose tissue, where they restore metabolic homeostasis, reduce inflammatory cell infiltration, and stimulate the secretion of beneficial insulin-sensitizing adipokines [122]. For this reason, a new class of drugs was developed to combine their therapeutic effects. Its leading representative is tirzepatide, which, in the randomized phase 2b SYNERGY-NASH clinical trial, proved to be highly effective and superior to placebo regarding MASH resolution without worsening liver fibrosis. Furthermore, tirzepatide achieved greater weight reduction and improved insulin sensitivity more effectively than a selective GLP-1R agonist, as additional GIP activation enhances fat oxidation and improves lipid storage in adipose tissue [123]. Despite the promising phase II results, confirming the drug’s efficacy in larger trials and evaluating its impact on long-term outcomes associated with liver disease progression remains necessary.

### 15.7. Dual GLP-1R/GCGR Agonists

Parallel to research on the GLP-1R/GIPR axis, dual Glucagon-Like Peptide-1 Receptor and Glucagon Receptor (GLP-1R/GCGR) agonists are being intensively developed and clinically evaluated. While the GLP-1R pathway is responsible for the previously described hepatoprotective effects, inhibition of lipogenesis, suppression of inflammation, and activation of the GCGR enhances fatty acid β-oxidation in the liver, limits fatty acid synthesis at the cellular level, and increases overall energy expenditure. Consequently, the combined action on both receptors leads to greater improvements in glucose metabolism, more effective body weight reduction, and more efficient liver fat clearance compared to GLP-1 monotherapy [124]. Survodutide is a structurally modified glucagon molecule in which changes to the amino acid sequence induce high affinity for both receptors, with a slight bias toward GCGR. In a 48-week Phase II trial, the drug led to the resolution of MASH without worsening of fibrosis in up to 62% of patients, as well as at least a 30% reduction in liver fat content in the majority of subjects. Improvement in liver fibrosis by at least one stage was observed in approximately one-third of participants receiving the drug, compared to 22% in the placebo group [125]. Pemvidutide, on the other hand, is a unique dual GLP-1R and GCGR agonist with a balanced 1:1 activation ratio. As a result, it effectively suppresses appetite while simultaneously increasing energy expenditure and enhancing hepatic fat burning, translating into greater weight loss and optimization of metabolic parameters compared to drugs with biased receptor activity. Treatment with pemvidutide over 12 weeks led to a 68.5% reduction in liver fat content, with more than half of the participants achieving complete normalization of liver fat levels. The drug also demonstrated potent anti-inflammatory activity in the liver, as evidenced by a significant decrease in serum ALT levels and a reduction in cT1 relaxation time below the safe threshold of 800 ms across all treated groups [126].

### 15.8. Triple GLP-1R, GIPR, and GCGR Agonists

Preclinical and clinical evidence indicates that single-molecule triple GLP-1R, GIPR, and GCGR agonists may offer greater efficacy in body weight reduction than dual agonists while maintaining a similar safety and tolerability profile. An example of such a compound is retatrutide, which exhibits stronger affinity for the GIPR compared to the natural GIP hormone, whereas its affinity for the GLP-1R and GCGR is weaker than that of their endogenous counterparts [113]. The use of retatrutide in adult patients with obesity or overweight and associated weight-related complications led to a significant decrease in liver fat content, reaching up to 86% at 48 weeks, which allowed for complete resolution of steatosis in over 85% of patients receiving the highest doses. This effect was closely linked to an approximately 20% reduction in body weight, accompanied by improvements in insulin sensitivity, lipid profile, and biomarkers of liver fibrosis and hepatocyte apoptosis [127].

### 15.9. FXR Agonists

Farnesoid X Receptors (FXRs) are nuclear transcription factors located primarily in the liver and intestines, and are responsible for maintaining bile acid, lipid, and glucose homeostasis. Their activation inhibits bile acid synthesis in the liver through SHP protein-dependent mechanisms, protecting hepatocytes from toxic accumulation of these compounds. Additionally, FXR agonists improve insulin sensitivity, increase glycogen synthesis, and exert anti-inflammatory effects, in part by dampening procholestatic and proinflammatory immune responses. Due to these comprehensive actions, these molecules represent a promising therapeutic option for conditions such as primary biliary cholangitis and steatotic liver disease [128]. A prominent representative of this group is vonafexor, a second-generation non-steroidal FXR agonist, which, in a 12-week phase IIa trial, induced a strong reduction in liver fat content as well as significant improvements in liver enzymes and imaging biomarkers of fibrosis and inflammation. The therapy was accompanied by a meaningful reduction in body weight and waist circumference, alongside an unprecedented improvement in eGFR for this drug class. This indicates a favorable effect on renal function and provides a rationale for further investigation in patients with mild to moderate renal impairment and suspected MASH [113,129]. Conversely, obeticholic acid demonstrated promising results in a phase 3 trial, where its administration led to significant, dose-dependent improvement in liver fibrosis without worsening of MASH, while simultaneously reducing markers of liver injury. Although the drug induced moderate weight loss and improved histological features of steatohepatitis, its use was associated with adverse events, such as severe dose-dependent pruritus and atherogenic lipid profile alterations. Consequently, obeticholic acid is no longer under clinical development for MASH [130,131].

## 16. Research Directions and Perspectives

Despite the dynamic development of non-invasive diagnostic methods, developing biomarkers with sufficient sensitivity and specificity to allow for a precise assessment of MASH activity, the degree of fibrosis, and treatment response remains a significant challenge. Currently used non-invasive tests do not always enable accurate differentiation between individual disease stages, and replacing liver biopsy with reliable non-invasive markers continues to be an unmet clinical need. One promising candidate is thrombospondin-2 (TSP2), which may find future application as a biomarker supporting the identification of patients with advanced fibrosis and active MASH. Unlike markers reflecting solely the metabolic or biochemical consequences of the disease, TSP2 is associated with extracellular matrix remodeling and fibrogenesis, creating the potential to monitor fibrosis dynamics. Changes in TSP2 concentrations observed in relation to changes in disease severity also indicate its potential utility in evaluating treatment response. However, it should be emphasized that its true clinical value and position in diagnostic algorithms require further validation and direct comparison with currently used biomarkers and non-invasive methods for evaluating fibrosis [132,133].

Biosensors also represent a promising direction in the development of MASLD/MASH diagnostics, potentially serving as a valuable supplement or alternative to current disease assessment methods in the future. Modern microsensors could enable the rapid and minimally invasive detection of changes in the concentration of selected biomarkers, potentially allowing for the earlier identification of adverse metabolic and liver-related changes. Integrating these measurements with artificial intelligence analytics may further enhance data interpretation capabilities and enable more precise patient monitoring. Particularly promising are point-of-care devices, which could potentially allow for the rapid determination of multiple biomarkers related to metabolic, inflammatory, and fibrotic processes. However, these solutions remain at the stage of translational research, and their implementation into clinical practice requires confirmation of their accuracy, reproducibility, as well as diagnostic and prognostic value in large, diverse patient populations [134].

Future priorities should also include the further development and standardization of modern omics technologies for use in the diagnosis MASLD, which still face numerous barriers resulting from, among other things, technical limitations, a lack of unified data integration methods, and limited availability of appropriate infrastructure and representative research cohorts. One approach that can help reduce the gap between laboratory discoveries and their application in everyday clinical practice is machine learning. Machine learning algorithms enable the integration and analysis of multidimensional omics data, the identification of the most significant biological signals, and the construction of models supporting the diagnostics and stratification of patients with MASLD. A key role will be played by the external validation of models in independent and diverse populations, the assessment of their reproducibility and interpretability, as well as the determination of their actual clinical utility [135].

## 17. Conclusions

MASLD, previously known as non-alcoholic fatty liver disease, is a major global health problem that is strongly associated with obesity, insulin resistance, T2DM. It is no longer considered a liver-specific condition but rather a systemic metabolic disorder affecting multiple organ systems. The pathogenesis of MASLD is multifactorial (“multiple-hit hypothesis”) and involves insulin resistance, adipose tissue dysfunction, oxidative stress, mitochondrial dysfunction, alterations in the gut microbiota, genetic predisposition, and chronic low-grade inflammation. The disease may progress from steatosis to steatohepatitis, fibrosis, cirrhosis, and hepatocellular carcinoma, with fibrosis stage being the most important prognostic factor. Despite advances in non-invasive methods, liver biopsy remains the gold standard for diagnosis, although a stepwise clinical approach using non-invasive risk stratification is being increasingly applied. Early identification of high-risk patients is crucial, as hepatocellular carcinoma may develop even in the absence of cirrhosis. Cardiovascular disease is the leading cause of death in patients with MASLD, exceeding liver-related complications. This highlights the need for comprehensive management addressing comorbidities such as T2DM, chronic kidney disease, and obstructive sleep apnea. Lifestyle modification remains the cornerstone of therapy, which includes weight reduction, a Mediterranean diet, and increased physical activity. Selected pharmacological agents (pioglitazone, GLP-1 receptor agonists, SGLT-2 inhibitors, and vitamin E) show benefit in specific patient populations, while emerging therapies are still under investigation. Currently, no definitive disease-modifying treatment for MASLD has been approved. Research directions include developing molecular biomarkers and advanced imaging techniques, using artificial intelligence in histopathology analysis, and targeting gut microbiome modulation. These approaches require further validation before clinical implementation. In summary, MASLD is a multisystem disease requiring early diagnosis, risk stratification, and a comprehensive therapeutic approach supported by ongoing translational research.

## Data Availability

No new data were created or analyzed in this study. Data sharing is not applicable to this article.

## Abbreviations

The following abbreviations are used in this manuscript:

MASLD	Metabolic dysfunction-associated steatotic liver disease
NAFLD	Non-alcoholic fatty liver disease
MASH	Metabolic dysfunction-associated steatohepatitis
HCC	Hepatocellular carcinoma
PY	Person-years
T2DM	Type 2 diabetes mellitus
ALT	Alanine aminotransferase
AST	Aspartate aminotransferase
GGT	Gamma-glutamyl transferase
FIB-4	Fibrosis-4
NFS	NAFLD Fibrosis Score
APRI	aspartate aminotransferase to platelet ratio index
MELD	Model for End-Stage Liver Disease
VCTE	Vibration-controlled transient elastography
ARFI	Acoustic radiation force impulse
MRE	Magnetic resonance elastography
MRI-PDFF	Proton density fat fraction magnetic resonance imaging
PPAR-γ	Peroxisome proliferator-activated receptor γ
GLP-1RAs	Glucagon-like peptide-1 receptor agonists
SGLT-2	Sodium-glucose cotransporter 2
LDL	Low-density lipoprotein
FGF21	Fibroblast growth factor 21
HDL	High-Density Lipoprotein
SPPARMα	Selective PPAR-α modulator
GLP-1R/GIPR	Glucagon-Like Peptide-1 Receptor and Glucose-Dependent Insulinotropic Polypeptide Receptor
GLP-1R/GCGR	Glucagon-Like Peptide-1 Receptor and Glucagon Receptor
FXR	Farnesoid X Receptor
